# The Short-Stature Homeobox-Containing Gene (*shox*/*SHOX*) Is Required for the Regulation of Cell Proliferation and Bone Differentiation in Zebrafish Embryo and Human Mesenchymal Stem Cells

**DOI:** 10.3389/fendo.2017.00125

**Published:** 2017-06-08

**Authors:** Tomoaki Yokokura, Hiroyasu Kamei, Takashi Shibano, Daisuke Yamanaka, Rie Sawada-Yamaguchi, Fumihiko Hakuno, Shin-Ichiro Takahashi, Toshiaki Shimizu

**Affiliations:** ^1^Juntendo University Graduate School of Medicine, Bunkyo, Japan; ^2^Department of Animal Sciences, Graduate School of Agriculture and Life Sciences, The University of Tokyo, Bunkyo, Japan; ^3^Department of Applied Biological Chemistry, Graduate School of Agriculture and Life Sciences, The University of Tokyo, Bunkyo, Japan; ^4^Faculty of Natural System, Institute of Science and Engineering, Kanazawa University, Kanazawa, Japan; ^5^Department of Oncology and Pathology, Cancer Centre Karolinska, Karolinska Institutet, Stockholm, Sweden; ^6^Department of Veterinary Medical Sciences, Graduate School of Agriculture and Life Science, The University of Tokyo, Bunkyo, Japan

**Keywords:** *SHOX*, zebrafish embryo, human mesenchymal stem cells, proliferation, osteogenic differentiation

## Abstract

The short-stature homeobox-containing gene (*SHOX*) was originally discovered as one of genes responsible for idiopathic short-stature syndromes in humans. Previous studies in animal models have shown the evolutionarily conserved link between this gene and skeletal formation in early embryogenesis. Here, we characterized developmental roles of *shox*/*SHOX* in zebrafish embryos and human mesenchymal stem cells (hMSCs) using loss-of-function approaches. Morpholino oligo-mediated knockdown of zebrafish *shox* markedly hindered cell proliferation in the anterior region of the pharyngula embryos, which was accompanied by reduction in the *dlx2* expression at mesenchymal core sites for future pharyngeal bones. In addition, the impaired *shox* expression transiently increased expression levels of skeletal differentiation genes in early larval stage. In cell culture studies, we found that hMSCs expressed *SHOX*; the siRNA-mediated blockade of *SHOX* expression significantly blunted cell proliferation in undifferentiated hMSCs but the loss of *SHOX* expression did augment the expressions of subsets of early osteogenic genes during early osteoblast differentiation. These data suggest that *shox*/*SHOX* maintains the population of embryonic bone progenitor cells by keeping its proliferative status and by repressing the onset of early osteogenic gene expression. The current study for the first time shows cellular and developmental responses caused by *shox/SHOX* deficiency in zebrafish embryos and hMSCs, and it expands our understanding of the role of this gene in early stages of skeletal growth.

## Introduction

Leri–Weill dyschondrosteosis (LWD) is an inherited skeletal deformity accompanied by short stature, mesomelia, and Madelung wrist deformity (1). The LWD gene is linked to abnormalities in the pseudoautosomal region (PAR1) on the sex chromosomes, and to the short-stature homeobox-containing gene (*SHOX*). The *SHOX* gene resides within the PAR1 and was first discovered as a gene strongly linked with the LWD (2–4). The phenotypes in LWD are complex, but the major mark of the skeletal deformity is shortening of the zeugopods (5). The proliferative chondrocytes in the growth plates of the LWD patients’ fore-/hind-limb zeugopods are highly disrupted, and it is noteworthy that the zeugopodial *SHOX* expression in embryonic human limb buds is spatially corresponding to the defect in LWD patients (6–11). Since the most prominent feature of LWD is the defect in cartilages and bones, the primary action of the *SHOX* is likely exerted in chondrocytes, osteocytes, and potentially in their progenitor cells.

Bone formation is initiated during the embryonic/fetal period, and it continues until the time of adolescence. The chondrocytes and osteocytes play central roles in bone formation, and it is also well accepted that the mesenchymal stem cells (MSCs) are major skeletal progenitor cells of both these types of cells (12–14). The *SHOX* gene is indeed expressed in the embryonic limb bud, mesenchymal core of early pharyngeal tissues, and in hypertrophic chondrocytes of adolescent bone, suggesting crucial developmental role(s) of this gene in these cells (15, 16). Given the prominent expression of *SHOX* gene in early embryonic limb bud, it is plausible that *SHOX* is expressed in skeletal progenitor cells and controlling their cell fate thus governs bone development and skeletal growth. Despite the overt involvement of *SHOX* in early embryonic skeletal formation, to date, we have limited information about the developmental role of this gene. In addition, the *SHOX* gene does not exist in major rodent model species (such as mice and rat) (5, 17–19), which largely restricts *in vivo* studies on the developmental role of *SHOX* using rodent models.

Zebrafish is an ideal model species to study the role of *SHOX* gene in early embryogenesis due to its fast development and growth, easy manipulation of embryos and genes using well-established methods (20–23). Most importantly, *shox* presents in zebrafish genome and conserved between fish and human (24). In the previous study, we characterized the *shox* gene in zebrafish embryos and found that the impaired expression of *shox* resulted in hindered embryonic growth even before the bone mineralization became evident (25). These data suggested crucial roles of *shox* in early embryonic skeletal progenitor cells, but the molecular and cellular changes caused by *shox* deficiency were unclear. In addition, as a cell culture model, the human MSCs (hMSCs) with their established methodologies for differentiation provide an ideal system to analyze the role of genes in skeletal formation (12–14); however, the influence of *SHOX* deficiency in hMSCs has yet to be definitively determined. The major scope of the current study is to increase our understanding of the developmental roles of *shox*/*SHOX*. Toward this goal, we characterized the cellular and molecular changes induced by the *shox*/*SHOX* deficiency in the developing zebrafish embryo and hMSC models. Our data suggest that *shox/SHOX* regulates both mitogenic and differentiation events in skeletal progenitor cells in developing animals.

## Materials and Methods

### Materials

Chemicals and reagents were purchased from Wako (Tokyo, Japan) and Nacalai Tesque (Kyoto, Japan) unless noted otherwise. DNA ligase and restriction endonuclease were purchased from Promega (Madison, WI, USA). For reverse-transcription (RT)-PCR, Trizol reagent, M-MLV reverse transcriptase and oligonucleotide primers were purchased from Invitrogen Life Technologies (Invitrogen, Carlsbad, CA, USA). The translation block antisense-morpholino oligo (MO) was purchased from Gene Tools, LLC (Philomath, OR, USA). A human osteosarcoma cell line, U2OS cells, was obtained from the American Type Culture Collection (ATCC; HTB-96) (ATCC, Manassas, VA, USA).

### Zebrafish Husbandry

Wild-type zebrafish (*Danio rerio*) were maintained at 28°C on a 14-h:10-h (light:dark) cycle as previously described. Embryos were obtained from natural crossings and the fertilized embryos were grown in E3 embryo rearing solution (20) at 28.5°C according to the standard method (21). For the sampling, embryos were anesthetized in tricainemesylate (ethyl 3-aminobenzoate methanesulfonate; Sigma-Aldrich Japan, Tokyo). This study was carried out in accordance with the recommended guidelines of the committee of the Life Science Research Ethics and Safety in the Graduate School of Agriculture and Life Sciences at The University of Tokyo, and the Guide for the Care and Use of Laboratory Animals prepared by Kanazawa University.

### Microinjection Experiments

The translational block antisense MO against zebrafish *shox* mRNA and the standard control MO (*ctr* MO) were prepared by Gene Tools, LLC (Philomath, OR, USA) as previously reported (25), and specificity and efficiency of the *shox* MO were verified in that previous report. The MO-injected embryos were raised in E3 medium and were sampled at the determined time points.

### Whole-Mount Immunostaining and *In Situ* Hybridization Analyses

For the whole-mount staining analyses, specimens were fixed by transferring to 4% paraformaldehyde for a couple of hours to overnight. The whole-mount immunostaining was conducted using anti-phospho-Histone H3 (3377, Cell Signaling Technology Japan, Tokyo, Japan), anti-active-caspase-3 (559565, BD Pharmingen™, BD Biosciences Japan, Tokyo, Japan) and anti-myosin heavy chain (05-716, Millipore, Darmstadt, Germany) according to the instruction manuals. The anti-phospho-Histone H3 and anti-active-caspase-3 antibodies were further labeled with anti-Rabbit IgG conjugated with Alexa-546 or -596 as second antibody. The signals corresponding to the myosin heavy chain (MyHC) antibody were visualized by using anti-mouse IgG conjugated with Alexa488. The specimen was counterstained with Hoechst33342. To detect specific mRNA, the fixed specimens were subjected to the whole-mount *in situ* hybridization using specific cRNA probes for *dlx2* mRNA, *pax2a* mRNA, and *ntl* mRNA. The digoxigenin-labeled cRNA probes were prepared as previously described (26). Pictures were taken by Canon iVIS HF M52 (Canon, Tokyo, Japan) mounted on a stereomicroscope or by BZ9000 fluorescence microscope (Keyence, Tokyo, Japan).

### RT-PCR and Quantitative Real-time (qRT)-PCR Analyses

Total RNA was isolated from 10–25 zebrafish embryos or from more than 0.5 × 10^5^ culture cells by using TRIzol reagent (Invitrogen). Total RNA (1–2 µg) was reverse-transcribed to generate the first-strand cDNA using SuperScript II reverse transcriptase according to the manufacturer’s instructions. The qPCR analysis was performed using iQ SYBR Green Supermix (Bio-Rad, Hercules, CA, USA) or SYBR^®^ *Premix Ex Taq*™ II (Tli RNaseH Plus, TaKaRa, Shiga, Japan) according to the standard method. Standard curves were constructed from serial dilution of standard samples to analyzed relative amount of the target transcript in each specimen. The amount of particular gene transcript was calculated based on the standard curve and normalized to the β*-actin* mRNA level. The specificity of the PCR was verified by denaturing curve analysis. Primer sets used in RT-PCR and qRT-PCR are described in the Table 1.

**Table 1:** List of primer sequences used in quantitative real-time-PCR.

Name	Sequence (5′ → 3′)	Accession number
Zf *runx2b* F	ATGGCCGAGATCATCGCCGATCAC	NM_212862
Zf *runx2b* R	GCGGGCCACCTGGTTCTTCATAACC	

Zf *col10a*1 F	ATGGAACTACGAGTAGTAAGCATTCTT	NM_001083827
Zf *col10a1* R	TGGCAGACCTTCACCATCTTGTCCTG	

Zf *bmp2b* F	TCTCACGGTGCTGTTGCTCG	NM_131360
Zf *bmp2b* R	GATTTGCTTGGGGTGGGTTT	

Zf *ßactin* F	TATCATCTGCTTGTAACCCATTCTCT	NM_181601
Zf *ßactin* R	TCTGTCCCATACCAACCATGACA	

Hs *SHOX* F	CGGCCACTGCCCGGTGCATTT	NM_000451
Hs *SHOX* R	CACGTCCTCGCGCTTCTCTTTGC	

Hs *RUNX2* F	GCTCCGGAATGCCTCTGCTGTTATG	NM_001024630
Hs *RUNX2* R	GTGATAGGTAGCTACTTGGGGAGGA	

Hs *DLX5* F	GCGGCGCCTACAACCGCGT	NM_005221
Hs *DLX5* R	GCGGCCAGCTGAAAGCTGGAATA	

Hs *ALPL* F	GCTGTAAGGACATCGCCTACCAGCT	NM_000478
Hs *ALPL* R	CTCGTCACTCTCATACTCCACATCA	

Hs *ßACTIN* F	CACCCCGTGCTGCTGACCGA	NM_001101
Hs *ßACTIN* R	CAGGGATAGCACAGCCTGGATAGCA	

### Reporter DNA Constructs and Dual Luciferase Assays

To construct a luciferase reporter plasmid pLuc-NPPB1940, the previously reported 1,940 bp-long promoter sequences of the NPPB gene, which contains human SHOX binding sequences, were amplified by PCR using a specific primer set (5′-AAAGTCGACAAGCTTGCTTTTTGTAGAAA-3′, 5′-AAACCATGGGTCTCTGGAGGGACTGGG-3′) (27). The amplified DNA fragment was then subcloned into the 5′-upstream cloning sites of the firefly luciferase reporter gene in the pGL3-basic vector (Promega, Madison, WI, USA) using *Sal*I and *Nco*I sites. For testing the transfection efficiency, we used pRL-TK (Promega), which constitutively expresses the renilla lucierase under control of the thymidine kinase promoter. The hMSCs were transfected with 0.3 µg of pLuc-NPPB1940 in combination with 0.1 µg of pRL-TK by a polyethyleneimine-mediated lipofection method. The luciferase assay was carried out using the dual luciferase assay kit (Promega), and the firefly luciferase activities were normalized to the renilla luciferase activities.

### Cell Culture and siRNA Transfection

The cells were cultured at 37.0°C under the 5% CO_2_ condition. For the culture of U2OS cells, Dulbecco’s modified Eagle medium supplemented with 3.5 g/L glucose, penicillin 0.25 µg/mL, streptomycin 5 µg/mL, kanamycin 10 µg/mL, amphotericin-B, and 10% fetal bovine serum was used. Human mesenchymal stem cells (hMSCs) were obtained from a commercial dealer (Poietics™, Lonza Japan, Tokyo, Japan), and the cells were cultured at 37.0°C under the 5% CO_2_ condition. The hMSCs were cultured in MSC growth medium (MSCGM™, Lonza Japan) supplemented with MCGS (mesenchymal cell growth supplement cell culture tested, Lonza Japan), l-glutamine, and GA-1000 (Gentamicin sulfate, amphotericin-B, Lonza Japan). For the induction of osteogenic differentiation of hMSCs, the cells were kept in Differentiation medium BulletKits™—osteogenic (osteogenic basal medium supplemented with dexamethasone, l-glutamine, ascorbate, penicillin/streptomycin MCGS, β-glycerophosphate, Lonza Japan). The siRNAs targeting human SHOX mRNA (Sense: 5′-GUGGCACCCUACGUCAACAUG-3′; Antisense: 5′-UGUUGACGUAGGGUGCCACUC-3′) and the control siRNA (Sense: 5′-GUACCGCACGUCAUUCGUAUC-3′; Antisense: 5′-UACGAAUGACGUGCGGUACGU-3′) was synthesized by RNAi Inc. (Tokyo, Japan) and introduced to the hMSCs by standard protocol using RNAiMAX Reverse Transfections Lipofectamine^®^ reagent (Invitrogen).

### Immunoprecipitation (IP) and Immunoblotting (IB)

Immunoprecipitation was performed using 0.3 mg of protein from total cell lysate by conventional method using Protein G Sepharose 4 Fast Flow (GE Healthcare UK Ltd., England) according to the instruction manual. Immunoblot analyses were conducted as previously reported (25). The antibodies for SHOX (SAB2102136, Sigma, St. Louis, MO, USA), RUNX2 (#12556 D1L7F Rabbit mAb, Cell Signaling Technology Japan, Tokyo, Japan), DLX5 (ab109737, Abcam Japan, Tokyo, Japan), and Tubulin (#2148 α/β-Tubulin Antibody, Cell Signaling Technology Japan, Tokyo, Japan) were purchased from commercial dealers and used at indicated dilutions as manufacturer’s instruction. Total protein concentration was measured by Pierce™ BCA Protein Assay Kit (Thermo Fisher Scientific, Waltham, MA, USA), and equal amount of proteins were subjected to SDS-PAGE followed by IB analysis.

### DNA Synthesis Assay

To assess DNA synthesis of siRNA-transfected cells, EdU assay was performed using Click-iT^®^ Plus EdU Alexa Fluor^®^ 488 Imaging Kit(Thermo Fisher Scientific)according to the instruction manual. At the 3 day post transfection of the siRNA, the hMSCs were incubated with 10 µM of EdU (5-ethyl-2′-deoxyuridine, a nucleoside analog of thymidine) under GM conditions for 10 h for labeling the S-phase cells. After the EdU-detection reaction, cells were counterstained with Hoechst33342 for counting. More than 1.0 × 10^3^ cells were counted to calculate the rate of EdU-positive cell emergence in each experimental group.

### Cell Counting of hMSCs

To compare the cellular growth in the siRNA-transfected hMSCs, the cells were suspended in BD Trucount™ Tubes (BD Biosciences, Tokyo, Japan) and cell numbers were counted using a flow cytometer FACS Verse (BD Biosciences). The relative cell numbers were calculated as the cell numbers of day-0 group of siCTR introduced group is 1.0.

### Statistics

Student’s *t*-test was used to determine the statistical significance between two groups. Difference among multiple groups were determined by one-way analysis of variance, followed by the Tukey’s multiple comparison tests. Statistical calculation was performed using GraphPad Prism 6.0 software (GraphPad Software Inc., San Diego, CA, USA), and significance was accepted at *P* < 0.05.

## Results

### Blunted *shox* Expression Reduced Cell Proliferation of Developing Embryos

**Figure 1:**
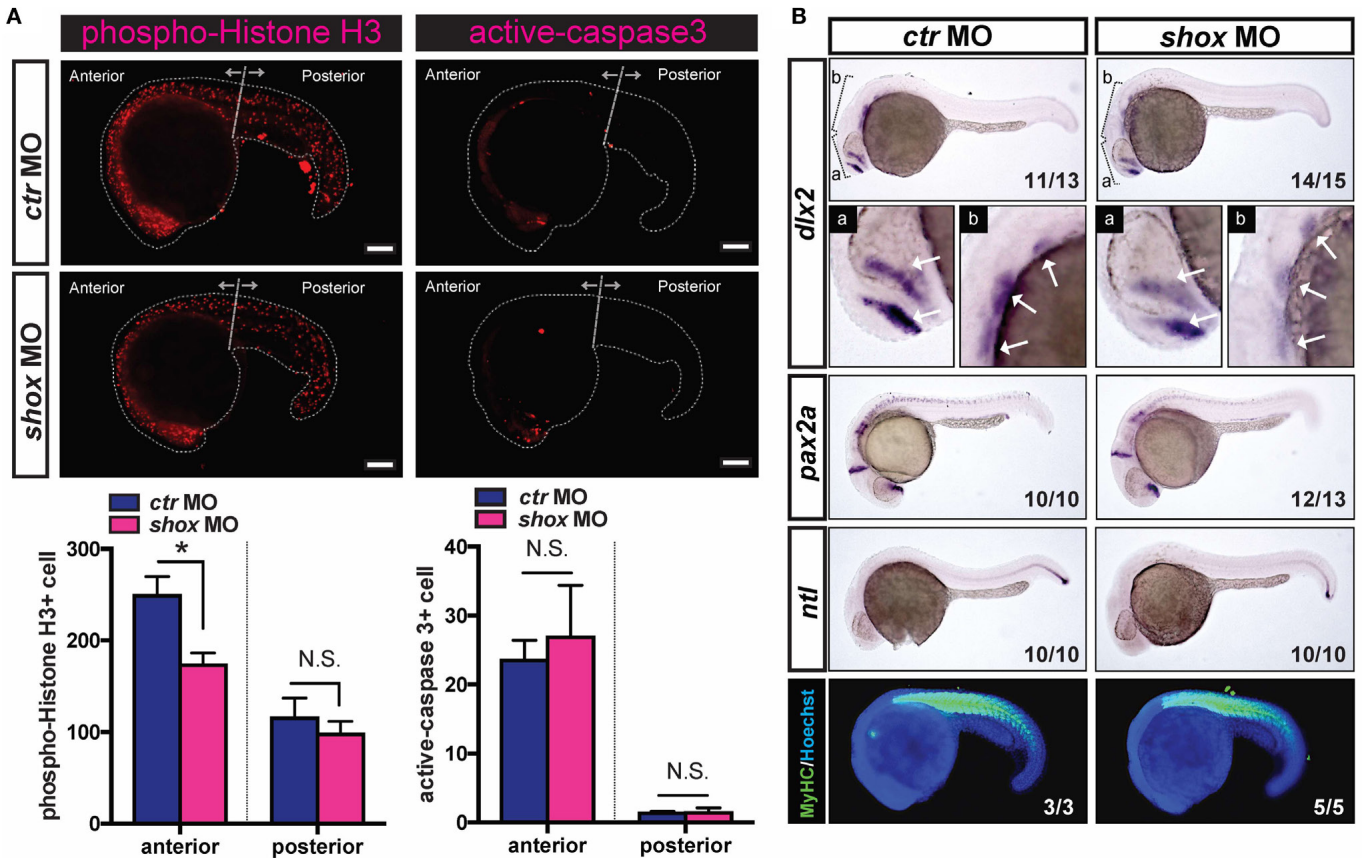
Translational blockade of zebrafish *shox* reduced cell proliferation and specific gene expression of skeletal progenitors in developing embryos. **(A)** Analyses of cell proliferation and cell death of *shox* morpholino oligo (MO) or control MO (*ctr* MO)-injected 22 hpf embryos. Bars, 100 µm. Representative results of phospho-Histone H3-staining and active-caspase-3-staining. Quantification data of phospho-Histone H3-staining of ctr MO-injected embryos (*n* = 5) and shox MO-injected embryos (*n* = 9). *P < 0.05. Quantification data of active caspase-3-staining of ctr MO-injected embryos (*n* = 3) and shox MO-injected embryos (*n* = 9). **(B)** Whole-mount *in situ* hybridization analysis and whole-mount immunostaining of 24 hpf embryos. The cRNA probe either for *dlx2* mRNA, *pax2a*, or *ntl* mRNA was used. The arrows indicate domains with *dlx2* positive signals. Whole-mount immunostaining analysis of myosin heavy chain (MyHC) (green) and Hoechst33342 (blue) was conducted. Representative results are displayed and the penetrance is shown in the lower right corner of each panel.

In a previous study, we showed the basic spatiotemporal developmental expression of *shox* assessed by quantitative PCR, *in situ* hybridization, and IB analyses, and, importantly, we also found that the loss of *shox* expression induced slightly reduced embryonic growth in early pharyngula stages (25). To test if the cell proliferation and cell death are controlled by early developmental *shox*, we analyzed the levels of phospho-Histone H3 as markers of cell proliferation and active caspase-3 as a cell death marker in *shox* MO or *ctr* MO-injected zebrafish embryos. As a result, the phospho-Histone H3 signals in *shox* MO-injected embryos were significantly reduced in anterior regions of the embryos compared to those in *ctr* MO embryos (Figure 1A, phospho-Histone H3, anterior). The effect of *shox* knockdown was not prominent in the posterior part of the body (Figure 1A, phospho-Histone H3, posterior). We then also analyzed active caspase-3 signals in both *shox* MO-injected embryos and *ctr* MO-injected embryos. Unlike the data for phospho-Histone H3, there were no obvious differences in active caspase-3 signals between the two groups (Figure 1A, active caspase-3). These data indicate that the *shox* participates in the cell cycle progression rather than the regulation of cell death in zebrafish embryo model.

### Impaired *shox* Expression Reduced dlx2 Expression in Pharyngula Stage Embryos

To investigate the effects of the blocked *shox* expression in embryonic development, the expression levels of several developmental marker genes were examined by whole-mount *in situ* hybridization. The *dlx2* gene is expressed in cells at the pharyngeal mesenchymal core, which contains the progenitor cells of the craniofacial skeletal bone. The *dlx2* mRNA signal levels were clearly reduced in *shox* MO-injected embryos (Figure 1B, *dlx2*). The majority of embryos had reduced *dlx2* mRNA level in the *shox* MO-injected group as indicated by its high phenotype penetrance (14/15). On the other hand, the expressions of *pax2a* (labeling subsets of neurons) and *ntl* (labeling posterior notochord) were also analyzed. Unlike the result for *dlx2*, in embryos with reduced *shox* expression neither *pax2a* nor *ntl* expression were dramatically changed (Figure 1B, *pax2a* and *ntl*). Furthermore, the effect of *shox* MO on muscle tissue development was investigated by staining the MyHC in developing embryos. As a result, the MyHC signals were not altered when *shox* expression was reduced (Figure 1B, MyHC), suggesting that the *shox* is not intimately involved in skeletal muscle development at early pharyngula stage.

### Knockdown of *shox* Transiently Increased Expression of Bone Differentiation Markers

**Figure 2:**
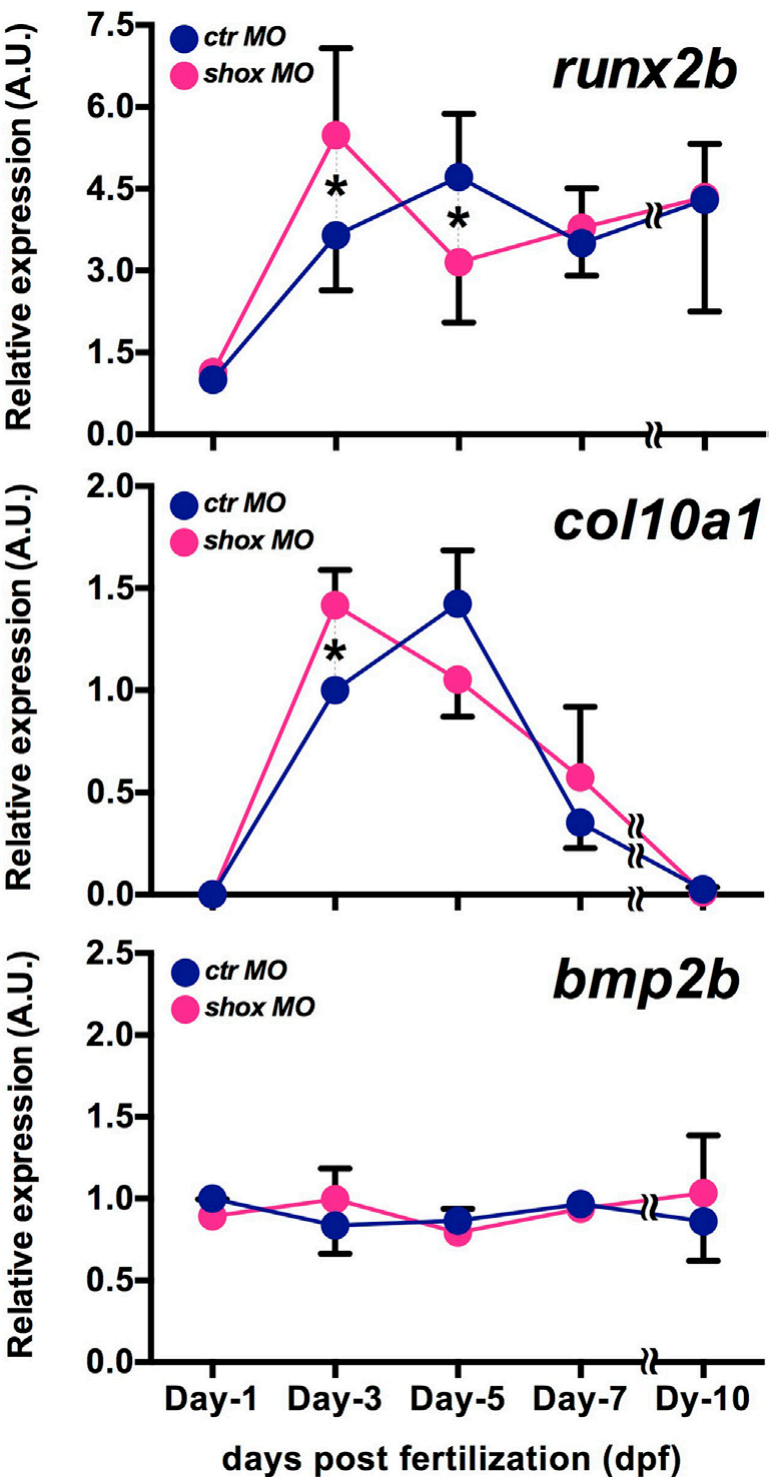
Translational blockade of zebrafish *shox* resulted in precocious expression of bone differentiation markers in developing embryos. Quantitative real-time-PCR analysis of bone differentiation markers (*runx2b, col10a1, bmp2b*) in morpholino oligo (MO)-injected embryos. Embryos were sampled at 1, 3, 5, 7, and 10 dpf. Transcript levels were normalized to the β*-actin* level, and the value is shown as relative abundance compared to that of control MO (*ctr* MO) at 1 dpf (*runx2b, bmp2b*) or at 3 dpf (*col10a1*). Data are mean ± SEM, 4–6 independent experiments. *P < 0.05.

Given the fact that the *shox* expression is increased in accordance with the developmental stage advances and that the loss of *shox* expression resulted in clearly reduced Shox protein level with defective bone ossification in our previous study (25), we have analyzed several bone differentiation marker genes in *shox* MO-injected embryos at 1, 3, 5, 7, and 10 days post fertilization. In *ctr* MO-injected embryos, the expression of *runx2b*, an osteogenic marker and *col10a1*, a chondrogenic marker, increased with embryonic growth. Expression levels of both genes peaked at 5 dpf, but in the case of *col10a1*, it declined thereafter (Figure 2, *runx2b* and *col10a1, ctr* MO). On the contrary, *shox* MO-injected embryos exhibited the increased *runx2b* and *col10a1* expression at earlier time points: the highest expression levels of these genes were marked at 3 dpf in *shox* MO-injected embryos (Figure 2, *runx2b* and *col10a1, shox* MO). The expression level of *bmp2b*, another bone differentiation-related gene, was not significantly changed at any sampling time point examined (Figure 2, *bmp2b, ctr* MO, *shox* MO). These data indicate that *shox* controls expression timing of a subset of genes involved in skeletal development during early larval period.

### *SHOX* Is Expressed in Human MSCs, and Its Expression Was Increased During Osteogenic Differentiation

**Figure 3:**
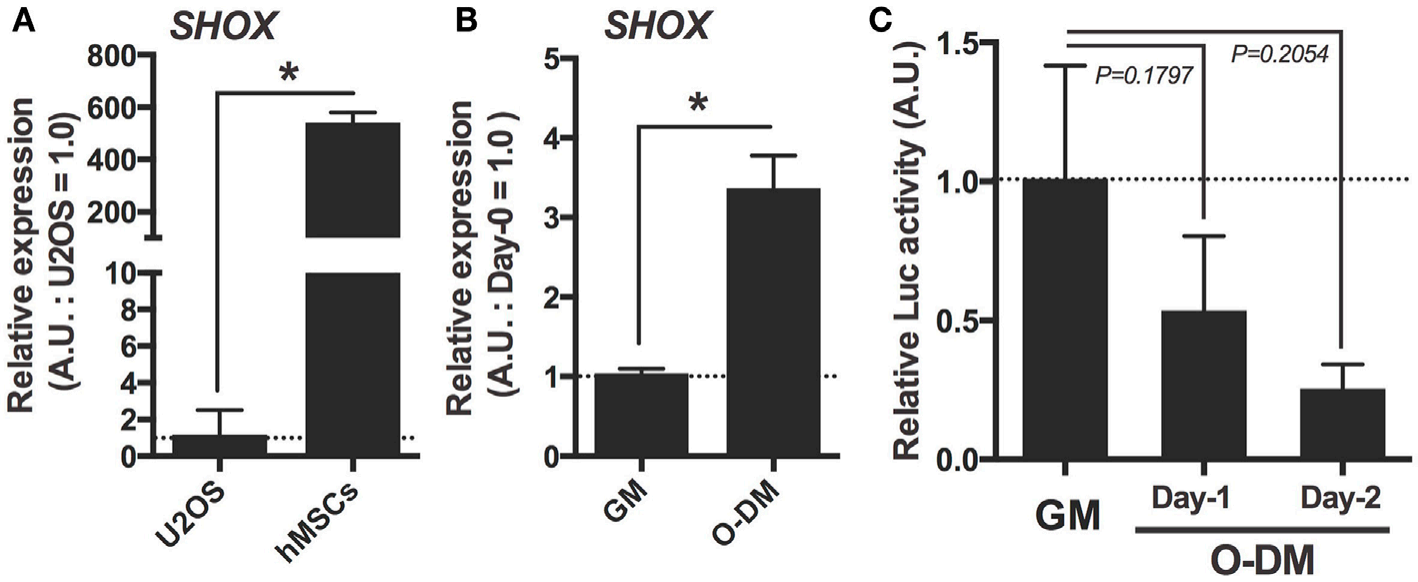
Expression and transcriptional activation activity of *SHOX* in human mesenchymal stem cells (hMSCs) under undifferentiated and under osteogenic differentiation conditions. **(A)** Quantitative real-time (qRT)-PCR analysis of *SHOX* gene in U2OS and undifferentiated hMSCs. Data are means of duplicated assays. Data are mean ± SEM, three independent experiments. *P < 0.05. **(B)** qRT-PCR analysis of *SHOX* gene in hMSCs under undifferentiated condition for 2 days or osteogenic differentiation condition for 7 days. Data are mean ± SEM, three independent experiments. *P < 0.05. GM: growth medium; O-DM: osteogenic differentiation medium **(C)** Relative luciferase activities of differentiating hMSCs transfected with luciferase reporter plasmid. At 12 h after transfection, the cells were collected and the luciferase activity was determined. Data are expressed as fold increase over the control group (cells not transfected pLuc-NPPB1940) in each experimental group. Data are mean ± SEM, 2–8 independent experiments.

Since the MSCs are indispensable for developing skeletal organs including cartilage and bone, and since our data showed a possibility that the expression of osteogenic differentiation marker genes were altered in *shox* knocked down zebrafish embryos, we focused on the role of this gene in hMSC. To begin with, expression levels of *SHOX* in hMSCs were measured by the qRT-PCR analysis. The U2OS cells whose *SHOX* expression level is known to be very low were used for comparison. Results demonstrated that the hMSCs had much higher *SHOX* gene expression (approximately 500 times higher) than that of U2OS cells (Figure 3A). This supports the appropriateness of the use of hMSCs for loss-of-function studies rather than the gain-of-function approach as usually applied in U2OS cells. We also examined the changes in *SHOX* gene expression level during osteogenic differentiation in a specific differentiation medium (osteogenic differentiation medium: O-DM). The *SHOX* expression was significantly increased by the 7-day long incubation in O-DM (Figure 3B). The change in endogenous *SHOX* activity was also tested by luciferase assay using the *NPPB* reporter construct that contains human SHOX binding sequences of *NPPB* gene promoter region as previously reported (27). The endogenous transcriptional activity of SHOX tended to be decreased within a few days post osteogenic differentiation compared to that in undifferentiated hMSCs (Figure 3C).

### siRNA-Mediated Knockdown of *SHOX* Expression Caused Reduced Cell Growth

**Figure 4:**
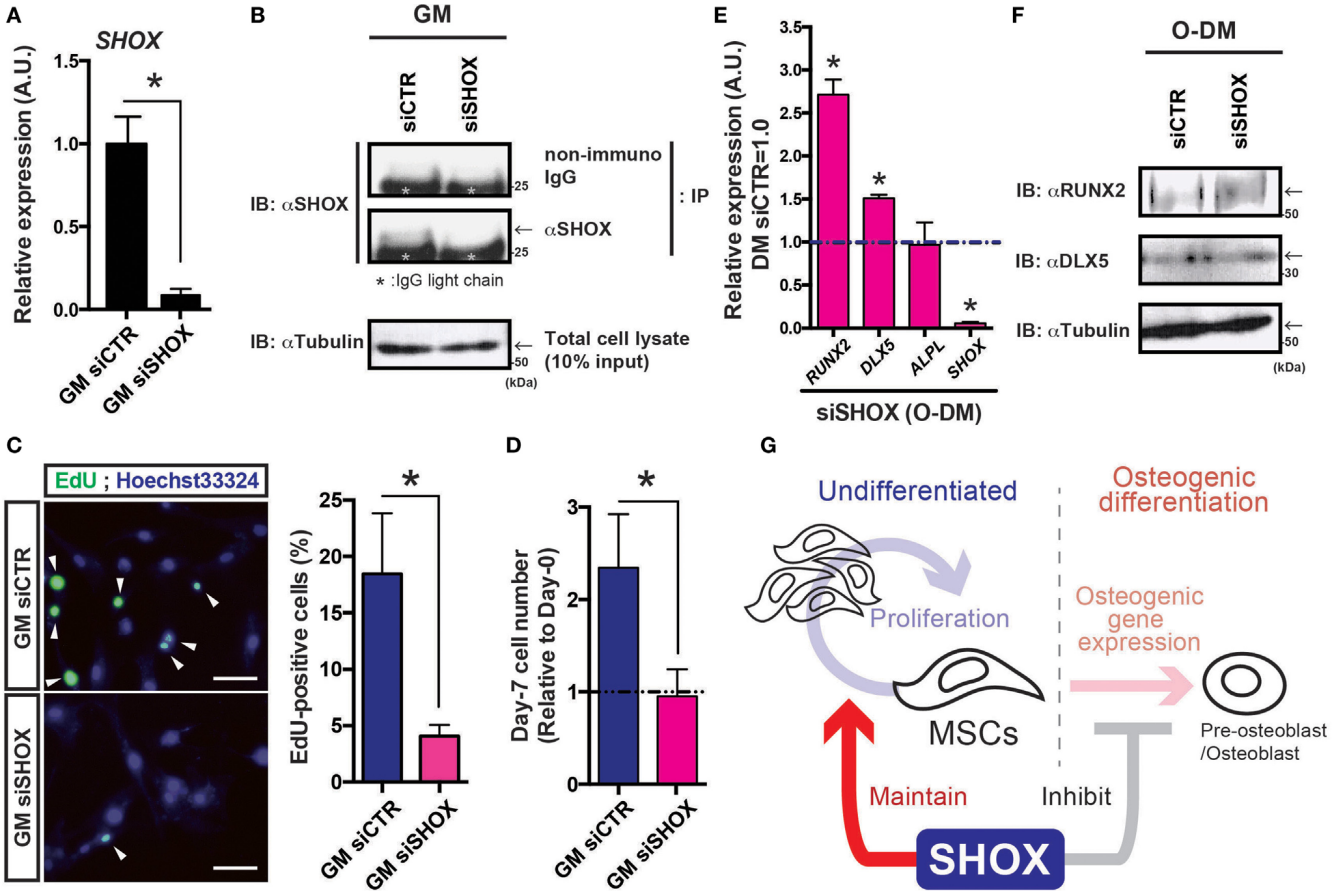
Reduced expression of *SHOX* hampered cell proliferation in human mesenchymal stem cells (hMSCs) under undifferentiated condition and disrupts osteogenic gene expression under differentiation-induction condition. **(A)** Changes in *SHOX* gene expression by siRNA in hMSCs. At the 4 days post siRNA transfection, total RNA was extracted from the cells and the levels of SHOX gene expression were examined by quantitative real-time (qRT)-PCR analysis. Data are mean ± SEM, three independent experiments. *P < 0.05. GM: growth medium. **(B)** Cell lysates were prepared at the 3 days post transfection of each siRNA and the changes in SHOX protein level are assessed by immunoprecipitation (IP) followed by immunoblotting (IB) using indicated antibodies/IgG. The IgG light chain is marked with asterisk. Arrows indicate the expected SHOX signal just above the IgG light chain. Tubulin was detected as an internal control. **(C)** The EdU assay was performed to test DNA synthesis of siRNA-treated cells under GM condition. Representative pictures and quantification data are shown. Arrowheads indicate EdU signals. Data are mean ± SEM, three independent experiments. *P < 0.05. **(D)** Changes in cell proliferation in undifferentiated hMSCs. The relative cell numbers are expressed by showing the value in initial (day-0) as 1.0, and the value in day-7 was compared. Data are mean ± SEM, three independent experiments. *P < 0.05. GM: growth medium **(E)** qRT-PCR analysis of *SHOX, ALPL, DLX5, RUNX2* genes in hMSCs under osteogenic differentiation condition. Data are mean ± SEM, three independent experiments. *P < 0.05. Undiffer., undifferentiated hMSCs; Osteo., hMSCs under osteogenic differentiation at day-4. **(F)** Cell lysates were prepared at the 4 days post transfection of each siRNA and the changes in protein levels of RUNX2, DLX5, and tubulin were assessed by IB using indicated antibodies. Arrows indicate the expected signal. **(G)** Schematic illustration of working hypothesis. The *shox*/*SHOX* expressed in mesenchymal stem cells (MSCs) maintains cell proliferation in early developmental period. On the other hand, during the osteogenic differentiation, *shox*/*SHOX* may coordinate the proper timing of the specific gene expression(s) required for differentiation process. Through these actions in MSCs, *shox*/*SHOX* regulates normal skeletal growth and development.

The hMSCs were transfected with siRNA against human SHOX mRNA and the expression levels of *SHOX* in hMSCs were evaluated by both qRT-PCR analysis and IB following the IP using SHOX antibody. As shown in Figure 4A, loss of the majority of *SHOX* transcripts in hMSCs was confirmed. Since we did not find clear SHOX protein signals in a simple IB analysis using a total cell lysates, we performed IP to concentrate the SHOX protein in the sample. After the IP–IB analysis, in agreement with the RNA levels, we found that the SHOX protein level in siSHOX group was clearly reduced from the one in siCTR group (Figure 4B). These data validate that the siRNA-mediated knockdown of SHOX worked efficiently in hMSCs. Since the *shox* MO-injected fish embryos showed reduced cell proliferation, we next tested the effect of impaired *SHOX* on cell proliferation under undifferentiated conditions (culture in growth medium: GM). First, the DNA synthesis was tested by the thymidine analog incorporation assay (EdU assay). In our assay, 15.0–24.6% of siCTR cells showed EdU-positive signals in 10 h in growth medium, but only 3.0–5.1% of siSHOX-treated cells showed EdU-positive signals (Figure 4C). Furthermore, we directly measure the cell number by using the flow cytometry-based cell counting method. In analogy with the results in EdU assay, the cell number of siCTR group was clearly increased by 7 days post transfection (2.4 times as many as initially), while the cells transfected with siRNA against SHOX (siSHOX) showed blunted cell growth (nearly no increase from initial) (Figure 4D). Since the loss of *shox* reduced embryonic cell proliferation and mesenchymal core gene expression (*dlx2*) and since hMSCs reduced its proliferation due to loss of *SHOX*, it is suggested that the *shox/SHOX* maintains skeletal mass via regulating proliferation of bone progenitor cells such as MSCs in a cell-autonomous manner.

### Impaired *SHOX* Expression Promoted the Osteogenic Differentiation

Furthermore, the results showing advanced profile of osteogenic gene expression in *shox* MO-injected zebrafish led us to analyze the role of *SHOX* during the osteogenic differentiation of hMSCs. The hMSCs treated with each siRNA were incubated in O-DM and the osteogenic genes expressions were tested at 4-day post O-DM incubation. The SHOX transcript at day-4 post transfection of the siRNA against SHOX significantly reduced its expression level (<1/20 of siCTR) in O-DM culture (Figure 4C, *SHOX*). Knockdown of *SHOX* resulted in significant gains in *RUNX2* and *DLX5* expressions, both of which are early osteogenic lineage marker genes (Figure 4E, *RUNX2, DLX5*). Expression levels of the other osteogenic gene such as *ALPL* were less affected by knockdown of *SHOX* under osteogenic differentiation condition (Figure 4E, *ALPL*). The protein levels of RUNX2 and DLX5 were also measured by IB experiment (Figure 4F). In agreement with the results from RNA quantification assay, the RUNX2 protein level was clearly increased in siSHOX group (1.45-fold increase from siCTR group), and the change in DLX5 protein level was 1.19-fold increase from siCTR group, by the data of two independent experiments. The increased *RUNX2* expression in siSHOX-treated hMSCs was consistent with zebrafish results, and all these results suggest that *shox/SHOX* cell autonomously inhibits early onset of osteogenic differentiation of growing skeleton.

## Discussion

Since the *SHOX* was discovered, their molecular and cellular functions have been studied in various models using human osteosarcoma U2OS cells, primary human oral fibroblasts, primary human chondrocytes, chicken micromass culture, and so on (8, 18, 19, 27–31). In this study, we examined roles of *shox*/*SHOX* in zebrafish embryos and in a human bone progenitor cell (hMSCs). We found that the blockade of *shox* expression caused blunted cell proliferation in pharyngula embryos with reduced expression of anterior bone progenitor marker gene such as *dlx2* (Figure 1). In addition, the reduced *shox* expression upregulated expression of bone differentiation marker genes in relatively earlier stages (Figure 2). The cell culture study demonstrated that the *SHOX* was expressed in undifferentiated hMSCs, and its transactivation activity tended to be reduced under osteogenic differentiation (Figure 3). The loss of *SHOX* expression clearly reduced both DNA synthesis and cell proliferation in undifferentiated hMSCs and it also augmented early osteogenic gene expression (Figure 4). All the data suggest that *SHOX* positively regulates the proliferation in the undifferentiated skeletal bone progenitor cells and negatively regulates early-stage osteogenic differentiation (Figure 4G). To our knowledge, it is the first time to examine the cellular and developmental changes caused by *shox/SHOX* deficiency in both early stage of developing animal and cultured MSCs. Our data would provide an insight for the deeper understanding of the role of *shox/SHOX* in skeletal growth and development.

In zebrafish experiments, the blunted *shox* expression reduced the cell proliferation in anterior region of pharyngula embryos, in conjunction with hindered expression of *dlx2*. Meanwhile, we failed to see obvious cell death in this stage of fish embryos induced by the loss of *shox* expression. Since the *dlx2* expressing cells are one of the major populations of future anterior skeleton (32), our current data imply that *shox* is required for maintaining skeletal progenitor cells. Indeed, current cell culture study using hMSCs showed that the hMSCs expressed *SHOX* and the siRNA-mediated *SHOX* knockdown affected the cell proliferation. Thus, data in both zebrafish embryo and hMSC models support the notion that *shox/SHOX* facilitates cell proliferation in embryonic skeletal progenitor cells. On the other hand, previous studies in U2OS osteosarcoma cells, human primary oral fibroblasts, and chondrocytes showed that overexpression of *SHOX* induced cell cycle arrest at G2/M phase and the activation of apoptotic pathway (8, 33). We presume the seemingly contradicting results between these previous studies and our current study would be due to the differences in experimental systems: previous studies took the gain-of-function approach in differentiated cell line, and the current one carried out the loss-of-function experiments of endogenously expressed *shox*/*SHOX* genes in embryonic cells/undifferentiated hMSCs. It is noteworthy that, in the case of the closely related *SHOX* paralog genes (*Shox2* in mice; *SHOX2* in human), overexpression and knockdown of these genes often did not reach the same conclusion: the knockout or knockdown of *Shox2* impaired cell proliferation but the overexpression of the gene never upregulated cell proliferation (19, 34). Alternatively, it is possible that *shox*/*SHOX* is required for cell proliferation in undifferentiated skeletal progenitor cells but not in the cells under differentiation or under fully differentiated status. Further study is needed to explain this issue, but it is plausible that the levels of *SHOX* expression and the cellular context cooperatively define its role in cell proliferation. Since the expression levels of *shox* increase during embryonic growth and development in zebrafish model, understanding the relationship between *shox*/*SHOX* expression level and its role in cell proliferation seems a very important issue to interpret the biological function of *SHOX* in skeletal formation.

Gene expression analysis showed the transient increase of both osteogenic and chondrogenic marker expressions in shox MO-injected embryos. Recent studies have demonstrated that the loss of mouse Shox2 resulted in accelerated chondrogenic differentiation of prechondrocyte and MSCs (30). These data encouraged us to analyze the role of shox/SHOX in skeletal progenitor cells. As we found in zebrafish model, the loss of SHOX expression in hMSCs also increased the expression of differentiation-related genes such as RUNX2 in early osteogenic differentiation. The mouse model of early onset of osteogenesis induced by precocious RUNX2 expression resulted in hindered skeletal growth likely due to the inappropriate calcification and abnormal chondrocyte differentiation (35). The early onset of chondrocyte differentiation is also linked with premature ossification of developing bone that restricts skeletal growth (36). Thus, the earlier elevation of differentiation genes (such as runx2b/RUNX2) would not be beneficial for the future skeletal growth in shox/SHOX deficient animals.

Previous studies showed that the fully differentiated osteoblast did not express prominent levels of *SHOX* and it suggests a less important role of *SHOX* in osteoblasts (5, 8). In our data, *SHOX* expressed in undifferentiated hMSCs and the loss of *SHOX* expression in hMSCs increased expression of early osteogenic marker genes (such as *RUNX2* and *DLX5*) under osteogenic differentiation condition. Given its increased expression and decreased transcription activity during early osteogenesis (as our results in Figure 3C), *SHOX* would act as a transcriptional repressor in differentiating osteogenic progenitor cells. Indeed, *SHOX* protein is known to behave not only as a transcriptional activator but also as a transcriptional repressor (19, 27, 29, 37). Currently, it is uncertain whether or not these osteogenic marker genes are the direct targets of *SHOX*. Investigations of its specific target genes in undifferentiated and differentiating hMSCs would be needed for delineating the developmental roles of this gene.

The *SHOX* family proteins are homeobox transcription factors, which often act as heterodimers to control target gene expressions (5). It might be supposed that the interacting partners of *SHOX* proteins are diverse depending on the cell type, cellular differentiation status, or environmental conditions, thus we see various, sometime opposite, functions of this protein in various experimental models. It is of great interest to identify binding partners of the *SHOX* protein in undifferentiated and/or differentiating skeletal progenitor cells such as MSCs. In addition to the bone and cartilage development, evidence from a recent study implies the potential link between the *SHOX2* expression and the specification of adipocytes, another important lineage originated from the MSCs (38). Furthermore, as well as its binding partners, we have been gradually developing greater understanding of the novel regulators of the *SHOX* family genes including microRNA (34). Since *shox*/*SHOX* is expressed in diverse types of cells and tissues (11, 25), it is likely that Shox/SHOX proteins have pleiotropic actions in many types of cells. Future studies on how the *shox/SHOX* expression and its action are regulated by other factors are doubtlessly required for comprehensive understanding of the role of this gene in animal growth and development.

## Ethics Statement

This study was carried out in accordance with the recommended guidelines of the committee of the Life Science Research Ethics and Safety in the Graduate School of Agriculture and Life Sciences at The University of Tokyo, and the Guide for the Care and Use of Laboratory Animals prepared by Kanazawa University.

## Author Contributions

TY, HK, TaS, DY, RS-Y, FH, S-IT, and ToS conceived and designed these experiments. TY, HK, TaS, DY, RS-Y, and FH performed the experiments. TY, HK, TaS, DY, RS-Y, and FH analyzed the data. HK, DY, FH, S-IT, and ToS contributed reagents and materials/analysis tools. TY, HK, TaS, FH, and S-IT wrote the paper.

## Conflict of Interest Statement

The authors declare that the research was conducted in the absence of any commercial or financial relationships that could be construed as a potential conflict of interest.

## Abbreviations

ISS, idiopathic short stature; LWD, Leri–Weill dyschondrosteosis; PAR1, the pseudo autosomal region 1; DIG, digoxigenin; hpf, hours post fertilization; dpf, days post fertilization.
